# Prevalence and sociodemographic correlates of common mental disorders among first-year university students in post-apartheid South Africa: implications for a public mental health approach to student wellness

**DOI:** 10.1186/s12889-019-7218-y

**Published:** 2019-07-10

**Authors:** Jason Bantjes, Christine Lochner, Wylene Saal, Janine Roos, Lian Taljaard, Daniel Page, Randy P. Auerbach, Philippe Mortier, Ronny Bruffaerts, Ronald C. Kessler, Dan J. Stein

**Affiliations:** 1 Department of Psychology, Stellenbosch University, Private Bag X1, Matieland, 7602 South Africa; 2 MRC Unit on Risk and Resilience in Mental Disorders, Department of Psychiatry, Stellenbosch University, Stellenbosch, South Africa; 3 MRC Unit on Risk and Resilience in Mental Disorders and Mental Health Information Centre of South Africa, Department of Psychiatry, Stellenbosch University, Stellenbosch, South Africa; 4 Division of Child and Adolescent Psychiatry, Columbia University, New York, USA; 5 Health Services Research Group, IMIM (Hospital del Mar Medical Research Institute), Barcelona, Spain; 6 CIBER Epidemiología y Salud Pública (CIBERESP), Madrid, Spain; 7 Universitair Psychiatrisch Centrum - Katholieke Universiteit Leuven (UPC-KUL), Campus Gasthuisberg, Leuven, Belgium; 8 Department of Healthcare Policy, Harvard Medical School, Boston, MA USA; 9 MRC Unit on Risk and Resilience in Mental Disorders, Department of Psychiatry and Mental Health, University of Cape Town, Cape Town, South Africa; 10 Research Group Psychiatry, Department of Neurosciences, KU Leuven University, Leuven, Belgium

**Keywords:** Mental health, Mental disorders, College students, University, South Africa, Social determinants of health

## Abstract

**Background:**

There is growing awareness of the need for effective prevention, early detection, and novel treatment approaches for common mental disorders (CMDs) among university students. Reliable epidemiological data on prevalence and correlates are the cornerstones of planning and implementing effective health services and adopting a public health approach to student wellness. Yet, there is a comparative lack of sound psychiatric epidemiological studies on CMDs among university students in low- and middle-income countries, like South Africa (SA). It is also unclear if historically marginalised groups of students are at increased risk for mental health problems in post-apartheid SA. The objective of the study was to investigate the prevalence and sociodemographic correlates of lifetime and 12-month CMDs among university students in SA, with a particular focus on vulnerability among students in historically excluded and marginalised segments of the population.

**Methods:**

Data were collected via self-report measures in an online survey of first-year students registered at two large universities (*n* = 1402). CMDs were assessed with previously-validated screening scales. Data were weighted and analysed using multivariate statistical methods.

**Results:**

A total of 38.5% of respondents reported at least one lifetime CMD, the most common being major depressive disorder (24.7%). Twelve-month prevalence of any CMD was 31.5%, with generalised anxiety disorder being the most common (20.8%). The median age of onset for any disorder was 15 years. The median proportional annual persistence of any disorder was 80.0%. Female students, students who reported an atypical sexual orientation, and students with disabilities were at significantly higher risk of any lifetime or 12-month disorder. Female gender, atypical sexual orientation, and disability were associated with elevated risk of internalising disorders, whereas male gender, identifying as White, and reporting an atypical sexual orientation were associated with elevated risk of externalising disorders. Older age, atypical sexual orientation, and disability were associated with elevated risk of bipolar spectrum disorder.

**Conclusions:**

Despite advances to promote greater social inclusion in post-apartheid SA, students who identify as female, students with atypical sexual orientations, and students with disabilities are nonetheless at increased risk of CMDs, although students who identify as Black and first-generation students are not.

**Electronic supplementary material:**

The online version of this article (10.1186/s12889-019-7218-y) contains supplementary material, which is available to authorized users.

## Background

Considerable research suggests that rates of psychopathology are higher among university students than the general population in a number of high income countries (HICs) [1, 2], with one-third of students typically reporting a common mental disorder (CMD) in the preceding 12 months [3, 4]. Mental illness has a profound and deleterious effect on social adjustment to university life [5, 6], impeding academic attainment, and leading to adverse health outcomes including death by suicide [7–9]. Based on these observations, there is a growing awareness of the need for effective prevention, early detection, and novel treatment approaches for CMDs among university students within a public mental health paradigm [9, 10]. Reliable epidemiological data on prevalence and correlates are the cornerstones of planning effective health services and implementing public mental health interventions. However, there is a comparative lack of sound psychiatric epidemiological studies among university students in many low- and middle-income countries (LMICs), including South Africa (SA).

The transition to university can be stressful, precipitating psychological distress and symptoms of psychopathology [7, 11, 12]. Entering university typically entails leaving home, adapting to a new social environment, increased academic pressure, greater opportunities for substance misuse, and financial pressure. This transition often coincides with the emergence of psychopathology [13, 14], as pre-existing mental health problems are exacerbated or new symptoms emerge in response to novel environmental stressors [8, 15, 16]. Consistent with these observations, data collected in 21 countries as part of the World Health Organisation (WHO) World Mental Health Surveys indicated that an average of 20.3% of college students across countries had 12-month DSM-IV disorders; 83.1% of which had pre-matriculation onsets [3]. Mood disturbances and symptoms of anxiety are the most common mental health problems reported by students. Twelve-month prevalence of major depressive disorder (MDD) and generalised anxiety disorder (GAD) estimates of 11.9% and 8.4% were found among students in the United States (US) (*n* = 287) [17], with similar rates of 8.9% for MDD and 15.7% for GAD among students in France (*n* = 1723) [18]. A multi-national study from 30 predominantly LMICs found a 13.0% one-week prevalence of depressive symptoms among students (*n* = 23,073) [19], although considerably higher rates for one-week prevalence of moderate or severe depressive symptoms were found among students in Nigeria (32.2%, *n* = 820) and Kenya (41.3%, *n* = 923) [20, 21]. In a survey of undergraduate and postgraduate university students in SA (*n* = 1337), 11,2% of students reported moderate to severe symptoms of depression and 15.8% reported moderate to severe symptoms of anxiety, although the study did not assess whether or not students would meet criteria for a diagnosis of a depressive or anxiety disorder [22]. Comparatively less attention has been paid to describing the prevalence of bipolar spectrum disorders among university students, although studies suggest that the 12-month prevalence of bipolar mood disorder among university students globally is between 1.8 and 3.1% [3, 23]. There is a dearth of research investigating bipolar spectrum disorder among university students in SA.

Studies consistently report hazardous substance use and experimentation with illicit substances among university students [24]. A study of US undergraduate students (*n* = 2843) found marked rates of cigarette smoking in the past month (15.0%), binge drinking in the past 2 weeks (51.1%), and marijuana use in the past month (16.6%) [25]. Twelve-month prevalence rates of 2.2% for frequent (≥10 times/year) and 14.7% for infrequent (1–9 times/year) illicit drug use were found among students in 12 Southeast Asian countries (*n* = 7923) [24]. A multinational study of students from three Caribbean countries and eight African countries (*n* = 7017), found 12-month prevalence rates of 3.5 and 17.2% for frequent (≥10 times/year) and infrequent (1–9 times/year) illicit drug use [26]; in the sub-sample of South African university students (*n* = 622) the rates of frequent and infrequent drug use were 3.4 and 13.0%, respectively [26]. Although attention has been given to describing patterns of substance use on college campuses, comparatively less attention has been paid to establishing rates of substance use disorders (SUDs). A notable exception is a study which found a 8.1% 12-month prevalence of SUDs among French students at six universities (*n* = 1723) [18].

Student mental health problems have been associated with a range of sociodemographic factors, including lower socioeconomic status (SES), gender, and being a member of an ethnic minority, although some authors have found no statistically significant associations between sociodemographic variables and psychopathology in student populations [21, 27]. Female students are generally at higher risk for internalising disorders (such as mood, anxiety and eating disorders) [7, 28–30], while male students are at increased risk of externalising disorders (including substance use, and conduct and impulse control disorders) [31, 32]. Lower SES has been associated with increased risk for depressive symptoms [20], anxiety disorders [18] and illicit drug use [24].

Structural approaches to stratification and mental health affirm that groups in the lower rungs of social hierarchies tend to suffer greater psychological distress [33]; wherever differences are found in rates of psychopathology issues of power, marginalisation, and subjugation are never far behind [34]. This is a potentially important consideration in SA, where there is a political and social history of marginalisation and oppression. Although it is unclear how the mental health of students from historically marginalised groups may be compromised by enduring socio-political forces in post-apartheid SA, there is convincing evidence from HICs that students in marginalised positions and those who are subject to discrimination are at increased risk of developing mental health problems. For example, students who are members of ethnic minorities appear to be at increased risk of a wide range of CMDs [35–38], such as mood disturbances [39, 40], anxiety disorders [30, 41], illicit drug use, and problems with impulse control [31, 42]. But this evidence is not entirely consistent. For example, although ethnicity accounted for a significant proportion of the variance in both depressive symptoms and suicidal behaviours in surveys of US college students in some samples [35, 43], surveys in other US samples found no significant differences between rates of CMDs among ethnic minority and White college students [44] or found White students to be at higher risk than ethnic minorities of engaging in hazardous alcohol use [45] and illicit substance use [46]. The increased vulnerability to mental illness observed among marginalised and minority groups is often understood to be a function of socio-political forces which subjugate and disempower particular groups, and limit access to social capital and economic opportunities. Indeed experiences of racial discrimination have been consistently associated with poor mental health [47]. Discrimination for reasons other than racism, can also have adverse impacts on mental health; perceived ageism, sexism, ableism, classism and heterosexism have strong associations with psychological distress [48]. This idea is supported by studies which have found that students with atypical sexual orientations (i.e. those identifying as gay, lesbian, bisexual, asexual or questioning) [7, 28, 49–51], and gender nonconforming students [7, 49, 52, 53], are at increased risk of developing psychiatric symptoms.

Although the associations between mental health and both physical impairments and physical health are well established, this relationship has not been extensively investigated among students. Correlations have been found between lower subjective physical health ratings and symptoms of depression and anxiety among university students [30]. Understanding the vulnerability of students with chronic health problems to psychological distress is important in SA, where almost 25 years after the advent of democracy the country continues to experience systemic problems with provision of health services [54] and ongoing inequalities with access to health care [55].

Data from HICs suggest that first-generation students (i.e. students whose parents did not complete tertiary education) are at higher risk than other students for mental health problems [1, 56–58]. First-generation students typically report higher levels of depressive symptoms and stress compared with other students, and are less likely to access campus mental health services [56].

It is important to understand the potential psychiatric vulnerability of students who form part of marginalised and historically excluded groups, particularly given the transformations that have occurred in higher education and the moves that have been made to diversify student populations in SA. In spite of policies of inclusion and the considerable successes that have been achieved to transform the demographic profile of students at SA universities, it is possible that enduring socio-political forces continue to compromise the psychological wellbeing of historically marginalised individuals. It is within this context that we set out to investigate the prevalence and sociodemographic correlates of CMDs among first-year university students in SA as part of the World Health Organisation (WHO) World Mental Health Surveys International College Student Project [59]. We were particularly interested in socio-political determinants of student mental health and the extent to which historically disadvantaged and marginalised groups of students might be at increased risk of CMDs in post-apartheid SA.

## Methods

The aims of this study were to: (1) establish the prevalence and age of onset of lifetime and 12-month CMDs among first-year SA university students; (2) document the proportion of students with a lifetime disorder who continued to experience symptoms during the past year, and the proportional persistence (i.e., the percentage of lifetime years with symptoms of each disorder from the age-of-onset to the age when the survey was completed); and (3) investigate associations between CMDs and sociodemographic characteristics.

### Procedure

All first-year students at the University of Cape Town in 2017 and Stellenbosch University in 2015 and 2017 (*N* = 14,575), were invited via email to participate in an anonymous online self-report survey. A total of 1407 students completed the survey (participation rate = 9.7%). To be included in the study students had to be 18 years or older, and enrolled for the first time at University. Students who did not identify as either male or female (*n* = 4) and who did not disclose their disability status (*n* = 1) were excluded from data analysis because there were too few cases to enable a meaningful analysis of these subgroups.

### Measures

Items adapted from the Composite International Diagnostic Interview used in the World Mental Health Surveys (WMH-CIDI) [60] and various validated screening instruments were used to assess:

1. *Sociodemographic characteristics.* Participants were asked to report their age, parents’ level of education, whether they had a serious physical impairment (e.g., vision, hearing, and movement impairment), and whether they suffered from any chronic illnesses (e.g., asthma, diabetes, migraine, chronic pain disorder). They were also asked how they identified in terms of gender, population group, and sexual orientation. Age was coded into two groups: (1) under 21 years old; and (2) 21 years old and older. We identified students as ‘first-generation students’ if neither of their parents had completed tertiary education. We identified a participant as a student with a disability if they reported any serious physical impairment or chronic health problem. Gender was coded as: (1) male; or (2) female. Population group was coded as: (1) “Black”; or (2) “White”. We used a broad definition of “Black” to include students who identified as Black-African, Indian, and Coloured (an official term used in SA for population classification and census data). A broad definition of Black was used in order to identify all students from historically excluded population groups; the use of these categories was not intended to reify sociocultural constructs, but was used with the aim of investigating ongoing social and economic disparities with access to health care, education and employment opportunities in SA. Sexual orientation was coded as: (1) heterosexual; or (2) atypical sexual orientation (i.e. lesbian, gay, bisexual, asexual or questioning).
2. *Common mental disorders (CMDs)*: We assessed the lifetime and 12-month prevalence of MDD, GAD, bipolar spectrum disorder, AUD and drug use disorder (DUD), using items adapted from the EPI-Q Screening Survey [61], WMH-CIDI [60], and Alcohol Use Disorders Identification Test [62]. Caseness was determined using the procedure validated in the Army Study to Assess Risk and Resilience in Service Members (Army STARRS) [63], and replicated in the WHO World Mental Health Surveys. For each of the disorders assessed, participants were asked when they first experienced symptoms (age of onset) and how many years since the age of onset they had symptoms.

### Data analysis

Data were weighted by population group and gender and analysed with SPSS. We used single imputation to determine missing values for current AUD in the 2017 sample because questions about age of onset and current symptoms for AUD were erroneously omitted from the 2017 survey. We calculated prevalence estimates (95% CIs) for all 12-month and lifetime mental disorders assessed. Estimates of age of onset and the proportional persistence (i.e., the percentage of lifetime years with symptoms of each disorder from age of onset) are reported as median values with associated inter-quartile ranges. For each disorder we also reported estimates of the percentage of respondents with onset of symptoms prior to age 18 (this being the age which students typically complete high school in SA). An analysis of covariance, controlling for age of onset, using generalised linear models with a negative binominal distribution and a log link function was used to identify sociodemographic correlates of years with symptoms for any disorder.

Logistic regression models were used to identify the sociodemographic correlates of: (1) any lifetime disorder; (2) any 12-month disorder; and (3) 12-month prevalence among lifetime cases. Risk factors for any lifetime or 12-month CMD were identified from the results of the preceding logistic regression models, and were used to determine if the number of risk factors a participant was exposed to was associated with: (1) any lifetime disorder; (2) any 12-month disorder; and (3) 12-month prevalence among lifetime cases.

Finally, we used multivariate analysis to identify sociodemographic risk factors and establish if the number of risk factors a student is exposed to is associated with an increased likelihood of: (1) an internalising disorder (i.e. MDD or GAD); (2) bipolar spectrum disorder; or (3) an externalising disorder (i.e. AUD or DUD).

### Ethics

Ethical approval was obtained from the Health Science Research Ethics Committee of the University of Cape Town (Reference: 744/2015) and Stellenbosch University (Reference: N13/10/149). Permission to conduct the study was obtained from both universities. Participation in the study was entirely voluntary and participants provided informed consent electronically prior to completing the survey. Information about crisis and student counselling services were provided to all participants, as well as information about where to access emergency care if participants experienced distress completing the survey. All data were anonymised and securely stored.

## Results

### Sample characteristics

The sample (*n* = 1402) consisted primarily of students who identified as female (55.2%), White ( 58.6%), heterosexual (77.8%), and able-bodied (81.6%). The majority of the sample was under 21 years of age (92.3%), and were not first-generation students (80.3%).

### Prevalence of mental disorders

Lifetime and 12-month prevalence estimates are presented in Table 1. A total of 38.5% (95%CI = 35.9–41.1) of respondents reported at least one lifetime disorder, the most common of which was MDD. The 12-month prevalence of any CMD was 31.5% (95%CI = 29.1–34.0), with GAD being the most common. The prevalence of the other disorders assessed were comparatively low, with 12-month rates ranging from a high of 5.6% for AUD to a low of 1.0% for bipolar spectrum disorder. A total of 81.2% of students with lifetime disorders, reported that they currently met diagnostic criteria for a disorder which had started more than a year ago. The most persistent disorder was GAD. The median age of onset for any disorder was 15 years (IQR = 13–17), with MDD and AUD having the lowest median age of onset (15 years) and DUD the highest (17 years). A total of 84.8% of respondents reported that their disorders had onset before the age of 18 years. The median proportional annual persistence (i.e., the proportion of years with symptoms) for any disorder was 80.0% (95%CI = 75.0–85.0, IQR = 63.0–100.0), with bipolar spectrum disorder having the highest median number of years with symptoms since the age of onset.

**Table 1:** Prevalence rates, age of onset, and proportional persistence of common mental disorders among first year university students in South Africa (*n* = 1402)

	Lifetime Prevalence % (95% CI)	12-month Prevalence % (95% CI)	12-month Prevalence among lifetime Cases % (95%CI)	Age of Onset Median (95%CI) [IQR]	Proportional Persistence Median % (95%CI) [IQR]
Major depressive disorder	24.7% (22.4–27.0)	13.6% (11.9–15.5)	54.9% (49.5–60.2)	15 (15–16) [13–17]	66.7% (66.7–75.0) [33.0–89.1]
Generalised anxiety disorder	22.6% (20.4–24.9)	20.8% (18.7–23.0)	91.8% (88.2–94.6)	16 (15–16) [13–17]	80.0% (75.0–83.0) [60.0–100.0]
Bipolar spectrum disorder	1.2% (0.7–1.9)	1.0% (0.5–1.7)	81.3% (54.4–96.0)	16 (14–18) [14–18]	89.0% (67.0–100.0) [67.0–100.0]
Alcohol use disorder	6.1% (4.9–7.5)	5.6% (4.5–6.9)	86.% (73.7–94.3)	15 (15–16) [14–16]	75.0% (75.0–89.0) [67.0–100.0]
Substance use disorder	4.8% (3.7–6.1)	3.1% (2.3–4.2)	64.2% (51.5–75.5)	16 (16–18) [15–18]	67.0% (50.0–88.0) [40.0–100.0]

*95%CI* 95% confidence interval

*IQR* interquartile range

Proportional persistence of mental disorder is defined as the percentage of lifetime years with mental disorder symptoms from age of onset to age at the completion of the survey

The confidence interval for medians was constructed without any distribution assumptions. The actual coverage level may thus be greater than the specified level

In the analysis of sociodemographic predictors of the number of years with symptoms of a CMD, controlling for age, we found that the persistence of symptoms was associated with being over 21 years of age (χ^2^(6) = 7.43, *p* = 0.01). No associations were found between the number of years with symptoms and gender (χ^2^(6) = 0.02, *p* = 0.88), population group (χ^2^(6) = 0.05, *p* = 0.82), being a first-generation student (χ^2^(6) = 3.02, *p* = 0.08), sexual orientation (χ^2^(6) = 0.05, p = 0.82), or disability status (χ^2^(6) = 2.65, *p* = 0.10).

### Sociodemographic correlates of common mental disorders

The results of the regression analysis of sociodemographic factors associated with a CMD are presented in Table 2. Identifying as female, reporting an atypical sexual orientation, and having a disability were risk factors for any lifetime disorder, any 12-month disorder, and the persistence of symptoms. No significant interactions were identified between risk factors.

**Table 2:** Multivariate analysis of sociodemographic predictors of any mental disorder among first year university students in South Africa (n = 1402)

	Predictor Distribution in total sample %	Any lifetime disorder aOR (95%CI)	Any 12-month disorder aOR (95%CI)	12-month Prevalence among Lifetime Cases aOR (95%CI)
Gender (female)	55.2	1.58 (1.26–1.98)*	1.75 (1.38–2.22)*	1.70 (1.33–2.16)*
Population group (Black)	41.4	0.97 (0.77–1.23)	0.87 (0.68–1.12)	0.89 (0.69–1.15)
Age (21 years or older)	7.7	1.29 (0.86–1.93)	1.20 (0.79–1.84)	1.18 (0.77–1.82)
First generation student	19.7	1.01 (0.75–1.36)	1.08 (0.79–1.48)	1.09 (0.79–1.50)
Sexual orientation (Atypical)	22.2	1.65 (1.26–2.16)*	1.65 (1.25–2.18)*	1.60 (1.20–2.12)*
Disability	18.4	1.61 (1.22–2.12)*	1.56 (1.17–2.07)*	1.54 (1.15–2.05)*
		R^2^ = 0.043	R^2^ = 0.047	R^2^ = 0.043
		X^2^(6) = 45.17	X^2^(6) = 47.98	X^2^(6) = 42.22
		p = 0.00	*p* = 0.00	p = 0.00

*aOR* adjusted odds ratio

*95%CI* 95% confidence interval

*Significant findings are indicated in bold* (α =0.05)

Table 3 shows prevalence estimates of any CMD among students who reported zero, one, two or three risk factors and the associations of level of risk with any lifetime and 12-month CMD, and 12-month prevalence of any CMD among lifetime cases. Exposure to two or more risk factors (i.e. identifying as female, reporting an atypical sexual orientation, or reporting a disability) was associated with significantly elevated odds of any lifetime or 12-month disorder as well as 12-month persistence among lifetime cases.

**Table 3:** Prevalence rates and predictors of lifetime, 12-month and 12-month prevalence among lifetime cases for any mental disorder by number of risk factors among first year university students in South Africa (n = 1402)

	Predictor Distribution in total sample %	Prevalence rates			Odds Ratios for number or risk factors as predictors of prevalence		
		Prevalence of any lifetime disorder for each level of risk %	Prevalence of any 12-month disorder for each level of risk %	12-month Prevalence among Lifetime Cases for any disorder for each level of risk %	Any lifetime disorder OR (95%CI)	Any 12-month disorder OR (95%CI)	12-month Prevalence among Lifetime Cases OR (95%CI)
0 risk factors	29.7	27.6	20.6	20.6	0.50 (0.39–0.65)*	0.46 (0.35–0.60)*	0.51 (0.39–0.66)*
Exactly 1 risk factor	47.7	39.7	32.7	30.8	1.10 (0.89–1.37)	1.12 (0.89–1.40)	1.07 (0.86–1.35)
Exactly 2 risk factors	19.7	47.7	41.5	38.8	1.61 (1.23–2.09)*	1.73 (1.32–2.27)*	1.64 (1.25–2.16)*
Exactly 3 risk factors	2.9	67.5	55.0	53.7	3.45 (1.76–6.74)*	2.75 (1.46–5.18)*	2.80 (1.50–5.22)*

*OR* odds ratio

*95%CI* 95% confidence interval

*Significant findings are indicated in bold* (α =0.05)

Risk factors: female gender, atypical sexual orientation, disability

The results of the multivariate analysis of sociodemographic predictors of lifetime and 12-month internalising disorders, bipolar spectrum disorder and externalising disorders are presented in Table 4. Increased likelihood of a lifetime internalising disorder was associated with female gender (aOR = 1.84, 95%CI = 1.46–2.33), atypical sexual orientation (aOR = 1.74, 95%CI = 1.32–2.29), and disability (aOR = 1.56, 95%CI = 1.17–2.07). An increased likelihood of lifetime bipolar spectrum disorder was associated with being over the age of 21 (aOR = 6.47, 95%CI = 1.96–21.36), atypical sexual orientation (aOR = 5.27, 1.85–15.03), and disability (aOR = 5.99, 95%CI = 2.12–16.98). Lifetime externalising disorders were associated with being male (aOR = 1.51, 95%CI = 1.05–2.17), identifying as White (aOR = 1.79, 95%CI = 1.17–2.70), and having an atypical sexual orientation (aOR = 1.67, 95%CI = 1.09–2.55).

**Table 4:** Multivariate analysis of sociodemographic predictors of internalising disorders, bipolar spectrum disorder, and externalising disorders among first year university students in South Africa (n = 1402)

	Predictor Distribution in total sample %	Internalising disorders			Bipolar spectrum disorder			Externalising disorders		
		Lifetime aOR (95%CI)	12-month aOR (95%CI)	12-month prevalence among lifetime cases aOR (95%CI)	Lifetime aOR (95%CI)	12-month aOR (95%CI)	12-month prevalence among lifetime cases aOR (95%CI)	Lifetime aOR (95%CI)	12-month aOR (95%CI)	12-month prevalence among lifetime cases aOR (95%CI)
Gender (female	55.2	1.84 (1.46–2.33)*	1.89 (1.47–2.45)*	1.36 (0.85–2.16)	0.61 (0.22–1.72)	0.50 (0.16–1.56)	0	0.66 (0.46–0.95)*	1.18 (0.79–1.77)	1.49 (0.73–3.07)
Population group (Black)	41.4	1.07 (0.84–1.37)	0.91 (0.70–1.18)	0.60 (0.38–0.96)*	1.01 (0.34–2.98)	0.86 (0.25–2.89)	0	0.57 (0.37–0.86)*	0.71 (0.46–1.11)	1.01 (0.43–2.38)
Age (21 years or older)	7.7	1.14 (0.75–1.73)	1.08 (0.69–1.70)	0.82 (0.37–1.81)	6.48 (1.96–21.39)*	3.32 (0.77–14.27)	0	1.71 (0.95–3.08)	1.41 (0.71–2.79)	0.93 (0.31–2.78)
First generation student	19.7	1.05 (0.77–1.43)	1.16 (0.84–1.61)	1.43 (0.80–2.56)	0.33 (0.08–1.44)	0.57 (0.13–2.59)	0	0.63 (0.36–1.11)	0.67 (0.37–1.22)	0.89 (0.31–2.62)
Sexual orientation (Atypical)	22.2	1.73 (1.32–2.28)*	1.68 (1.26–2.24)*	1.15 (0.70–1.91)	5.28 (1.85–15.07)*	4.02 (1.28–12.64)*	0	1.67 (1.09–2.55)*	1.78 (1.13–2.80)*	1.36 (0.61–3.06)
Disability	18.4	1.56 (1.17–2.07)*	1.39 (1.03–1.88)*	0.87 (0.52–1.45)	5.99 (2.12–16.97)*	6.62 (2.14–20.45)*	0	1.05 (0.66–1.69)	1.40 (0.87–2.24)	1.70 (0.65–4.43)
		R^2^ = 0.057	R^2^ = 0.048	R^2^ = 0.021	R^2^ = 0.150	R^2^ = 0.117		R^2^ = 0.036	R^2^ = 0.021	R^2^ = 0.035
		X^2^(6) = 58.41	X^2^(6) = 46.95	X^2^(6) = 6.52	X^2^(6) = 25.13	X^2^ (6) = 16.88		X^2^(6) = 24.15	X^2^ (6) = 12.24	X^2^ (6) = 3.51
		p = 0.00	p = 0.00	*p* = 0.37	*p* = 0.00	*p* = 0.01		*p* < 0.00	*p* = 0.06	*p* = 0.74

*aOR* adjusted odds ratio

*95%CI* 95% confidence interval

*Significant findings are indicated in **bold** (α =0.05)

A significantly increased risk of lifetime internalising disorder was associated with exposure to two risk factors (OR = 1.67, 95%CI = 1.28–2.19), and exposure to three risk factors (OR = 4.24, 95%CI = 2.17–8.29). The likelihood of reporting a bipolar spectrum disorder was associated with increased risk of exposure to two risk factors (OR = 11.61, 95%CI = 4.22–31.93). Exposure to two risk factors was associated with increased odds of an internalising disorder or a bipolar spectrum disorder (OR = 1.89, 95%CI = 1.32–2.71). An increased risk of reporting a lifetime externalising disorder was associated with exposure to two risk factors (OR = 1.89, 95% CI = 1.32–2.71). Detailed results of the analysis of level of risk associated with these disorders are available as supplementary material (Additional file 1).

## Discussion

This study is the first of its kind to report prevalence and sociodemographic correlates for a range of CMDs in a large sample of first-year university students in SA. An additional advantage is that we used well-validated instruments that allow for cross-national comparisons. The findings highlight the marked prevalence of mental health problems among SA students and show that the lifetime prevalence (38.5%) and 12-month prevalence (31.5%) for any CMD are higher than the 30% and 17% found among a nationally representative sample of the country’s general population [64]. These prevalence rates are broadly consistent with, although slightly higher than, those found among university students in other parts of the world [3, 4], confirming the need for an international focus on student mental health. Given the prevalence of CMDs among university students in SA, it would seem to be appropriate to adopt a public mental health approach to the promotion of student wellness. A public mental health approach would entail ongoing monitoring of the prevalence of CMDs on SA university campuses, the use of accurate epidemiological data to plan and evaluate services, and careful consideration of ecological and systemic factors which may compromise students’ mental health. It is significant that the median age of onset for any disorder was 15 years and that approximately 85.0% of disorders had their onsets during high school, as this highlights the fact that most mental health problems experienced by first-year university students pre-date their entry to university. This finding is consistent with previous studies which show that mental disorders typically have their onset during mid-adolescence [3, 13]. Any efforts to promote the mental health of university students in SA will need to include school-based programmes and improved access to adolescent psychiatric services.

It is noteworthy that the most common disorders, consistent with the results of studies of university students in other high income countries [3, 23], are MDD and GAD, highlighting the need for targeted interventions to address symptoms of depression and anxiety among SA university students. These findings are also consistent with previous studies highlighting the marked prevalence of symptoms of depression and anxiety among university students in SA [22] and other parts of Africa [20, 21]. Given the large number of students with GAD and MDD, it seems unlikely that conventional treatment approaches that rely on one-to-one psychotherapy and face-to-face counselling will be a feasible or affordable means of addressing this problem. Exploring the acceptability, efficacy and cost-effectiveness of alternative sustainable approaches, such as the use of group therapy and/or internet-based psychotherapy warrants careful investigation. Guided internet-based interventions, in particular, appear to be as effective as face-to-face psychotherapy in treating depression, anxiety and substance use [65–67], but as yet there are no published studies of the use of e-interventions among university students in SA. Studies are needed to establish the acceptability to university students of these alternative modes of delivering mental health interventions, the affordability and sustainability of these interventions, and their effectiveness.

We did not find any significant associations between population group and mental health status in our data. This finding is interesting in the light of contemporary discourses about the vulnerability of Black students and the challenges to achieve racial transformation at universities in post-apartheid SA [68–70]. Our data suggest that population group may be too crude a variable to be meaningfully employed in either the analysis of student mental health data or the planning of public mental health interventions on SA university campuses. This finding will, however, need to be validated in future studies which draw on larger more representative samples of students from across the country.

It is significant that we found gender to be associated with increased risk of CMDs. Gender is strongly associated with physical and mental health status, and exerts a significant influence on help seeking [71, 72]. Significant gender differences have been found in the physical and mental health status of American students [73, 74]. In spite of growing awareness of gender imbalances in health this issue has, until recently, received comparatively little attention in the mental health literature generally [34] and college mental health literature specifically. There is a particular paucity of studies on gender differences in the epidemiology of CMDs among students in LMICs, where issues of gender inequality are likely to be more marked than on campuses in western, high income, democratic countries. Gender-neutral approaches to mental health research are biased and ‘could contribute to a failure of health providers to deliver gender-sensitive mental health treatments and services, to the detriment of both men and women’ [75, 76]. Andermann has noted, ‘There is now growing evidence, from neuroscience to epidemiology and health services research, that investigations of gender differences in mental health can help us understand the aetiological determinants of mental disorders and lead to more tailored treatments for men and women’ [76] (p. 501). It is also noteworthy that we found students with atypical sexual orientations and students with disabilities to be at increased risk for mental illness, which is consistent with previous research in this area [7, 28, 49–51, 77, 78].

Female students in SA and those with atypical sexual orientations and disabilities might be at increased risk of CMDs for a number of reasons, including the possibility that: (1) they are disproportionately exposed to risk factors, such as interpersonal violence, sexual assault and trauma; (2) they face a higher number of social stressors and less social support than their heterosexual male able-bodied peers; and (3) they continue to be marginalised and experience themselves as being at the lower end of social hierarchies. While SA universities have become more inclusive and diverse since the advent of democracy, these institutions are still experienced by some students and faculty as maintaining gender norms which constrain women [79], and perpetuate historical inequalities on the basis of ancestry, class, disability status, and gender [70, 80, 81]. Although SA has a remarkably liberal constitution which protects the rights of women and LGBTQ individuals, there is still evidence of conservative gender roles and high levels of homophobia and homophobic violence in post-apartheid society [82, 83], and on local university campuses [81, 84]. Scholars have called attention to the silencing of queer voices in contemporary SA universities, and the lack of transformation in areas of sexual orientation and sexual identity [81, 85]. Future research might explore female, disabled, and LGBTQ students’ lived experience of oppression and marginalisation on SA university campuses, with a view to identifying potential opportunities to disrupt health-compromising oppressive practices where these exist.

Our data do not provide insight into the possible links between financial pressures and the mental health of university students in SA. This is an important line of inquiry especially given the attention that has been paid to the economic costs associated with enrolling in higher education in SA [86, 87], and the recent “fees must fall” protests which took place on university campuses across the country [88, 89]. It will be important for subsequent studies to take account of this and include reliable measures of socio-economic status, financial stress and economic factors which may compromise university students’ mental health.

The high rates of psychopathology found in our sample, strongly suggest that there is a need for well-resourced, accessible and sustainable student counselling services which provide evidenced-based treatments for CMDs on university campuses in SA. Our data also suggest that it would be appropriate to screen first-year students for CMDs and provide information about where to access treatment. However, screening for mental health problems is not without its limitations, particularly in low-resource environments where available treatment options may be inadequate [90]. It is also not always possible to reach at-risk students through screening surveys, which suggests that it may be appropriate to employ more targeted outreach to students who belong to two or more at-risk groups (i.e. students who identify as female, atypical sexual orientation or disabled). Such a strategy makes sense given that 41.5% of students with a current CMD report two of these risk factors, while 55.0% of students with a CMD report three risk factors.

Our findings do not provide insight into the level of role impairment among the high number of students with mental health problems nor the effects of these disorders on academic performance and retention. Subsequent studies in this area should document the impact of CMDs on social and academic function as this will help to determine priorities with respect to planning and funding student mental health services. It would also be important for subsequent studies to establish the proportion of students receiving psychological treatment and to describe potential barriers to treatment seeking, including attitudes to help seeking.

### Limitations

This cross-sectional study relied on self-report measures from a self-selected sample. The response rate was relatively low and the sample was drawn from only two well-resourced universities in the Western Cape Province of SA. These limitations restrict the generalisability of findings. It is also a limitation that we excluded 4 students who did not identify as either male or female, as this subgroup of gender non-conforming students was too small to analyse meaningfully.

## Conclusion

Our data add to the literature on the mental health care needs of university students and highlight the marked rates of psychopathology among first-year university students in SA. These data support the growing body of evidence that more attention needs to be paid to supporting the psychological wellbeing of young adults as they transition into tertiary education and highlight the need for a public mental health approach to promoting student welnness. Our findings strongly suggest that further investigation is warranted to understand the reasons for observed associations between CMDs and gender, sexual orientation and disability status. Understanding the reasons why specific groups of students are more vulnerable to mental illness has important implications for planning and delivering student mental health services, and for advancing our understanding of how social and political forces influence the mental health of students.

## Additional file

Additional file 1: **Table S1.** Prevalence rates and predictors of lifetime, 12-month and 12-month prevalence among lifetime cases for internalising disorder by number of risk factors among first year university students in South Africa (*n* = 1402). **Table S2.** Prevalence rates and predictors of lifetime, 12-month and 12-month prevalence among lifetime cases for bipolar disorder by number of risk factors among first year university students in South Africa (*n* = 1402). **Table S3.** Prevalence rates and predictors of lifetime, 12-month and 12-month prevalence among lifetime cases for externalising disorder by number of risk factors among first year university students in South Africa (*n* = 1402). (XLSX 13 kb)

## Data Availability

Due to ethical restrictions, the data cannot be made publicly available. The datasets used and/or analysed during the current study are available from the corresponding author on reasonable request.

## Abbreviations

95% CI: 95% confidence interval
aOR: Adjusted odds ratios
Army STARRS: Army Study to Assess Risk and Resilience in Service Members
AUD: Alcohol use disorder
CMD: Common mental disorder
GAD: Generalised anxiety disorder
HICs: High-income Countries
LGBTQ: Lesbian, gay, bisexual, transgender and queer or questioning
LMICs: Low- and middle-income countries
MDD: Major depressive disorder
OR: Odds ratios
SA: South Africa
SES: Socioeconomic status
SPSS: Statistical package for social sciences
SUD: Substance use disorder
US: United States
WHO: World Health Organisation
WMH-CIDI: Composite International Diagnostic Interview used in the World Mental Health Surveys
